# Characteristics and Outcomes of a Sample of Patients With COVID-19 Identified Through Social Media in Wuhan, China: Observational Study

**DOI:** 10.2196/20108

**Published:** 2020-08-13

**Authors:** Dong Liu, Yuyan Wang, Juan Wang, Jue Liu, Yongjie Yue, Wenjun Liu, Fuhai Zhang, Ziping Wang

**Affiliations:** 1 Renmin University of China Beijing China; 2 Key Laboratory of Carcinogenesis and Translational Research (Ministry of Education/Beijing) Department of Thoracic Medical Oncology Peking University Cancer Hospital & Institute Beijing China; 3 Department of Radiology Peking University Shougang Hospital Beijing China; 4 Department of Epidemiology and Biostatistics School of Public Health Peking University Beijing China; 5 School of Education Hebei Normal University Shijiazhuang China

**Keywords:** COVID-19, risk factors, web-based data, outcome, infectious disease, clinical characteristic, mortality, social media, prognosis, China, coronavirus

## Abstract

**Background:**

The number of deaths worldwide caused by coronavirus disease (COVID-19) is increasing rapidly. Information about the clinical characteristics of patients with COVID-19 who were not admitted to hospital is limited. Some risk factors of mortality associated with COVID-19 are controversial (eg, smoking). Moreover, the impact of city closure on mortality and admission rates is unknown.

**Objective:**

The aim of this study was to explore the risk factors of mortality associated with COVID-19 infection among a sample of patients in Wuhan whose conditions were reported on social media.

**Methods:**

We enrolled 599 patients with COVID-19 from 67 hospitals in Wuhan in the study; 117 of the participants (19.5%) were not admitted to hospital. The demographic, epidemiological, clinical, and radiological features of the patients were extracted from their social media posts and coded. Telephone follow-up was conducted 1 month later (between March 15 and 23, 2020) to check the clinical outcomes of the patients and acquire other relevant information.

**Results:**

The median age of patients with COVID-19 who died (72 years, IQR 66.5-82.0) was significantly higher than that of patients who recovered (61 years, IQR 53-69, *P*<.001). We found that lack of admission to hospital (odds ratio [OR] 5.82, 95% CI 3.36-10.1; *P*<.001), older age (OR 1.08, 95% CI 1.06-1.1; *P*<.001), diffuse distribution (OR 11.09, 95% CI 0.93-132.9; *P*=.058), and hypoxemia (odds ratio 2.94, 95% CI 1.32-6.6; *P*=.009) were associated with increasing odds of death. Smoking was not significantly associated with mortality risk (OR 0.9, 95% CI 0.44-1.85; *P*=.78).

**Conclusions:**

Older age, diffuse distribution, and hypoxemia are factors that can help clinicians identify patients with COVID-19 who have poor prognosis. Our study suggests that aggregated data from social media can also be comprehensive, immediate, and informative in disease prognosis.

## Introduction

In December 2019, a novel coronavirus disease (COVID-19) emerged in China and began to spread globally. As of April 08, 2020, the outbreak has resulted in 82,992 deaths worldwide [1]. Identification of early-stage clinical predictors of poor patient outcomes is essential to effectively prioritize resources for patients with the highest risks and lower death rates. Several case series from China and Italy have suggested that male sex, older age, hypertension, kidney disease, and myocardial injury are risk factors for severe COVID-19 [2-6]. Despite these studies, knowledge of the early stage risk factors associated with poor prognosis is still limited. In this paper, we present a series of cases reported on the internet (from 67 hospitals in Wuhan) with definite clinical outcomes (discharge or death as of March 30, 2020) and their early-stage characteristics (before hospital admission) to explore the early stage risk factors and clinical features of COVID-19 mortality.

## Methods

### Study Design

The study protocol was approved by the research ethics committee of Renmin University of China on February 5, 2020. Data were obtained from two sources: Weibo posts and a telephone survey. The Weibo data were posted on the internet by families impacted by COVID-19 between January 20 and February 15, 2020, and were collected between February 3 and February 15, 2020. Then, volunteers phoned each participant’s family to describe the study and obtain their oral consent to participate. Over 60% of the patients (599/911, 65.8%) agreed to participate and completed most of the questions. One month later, a follow-up telephone call was conducted to collect the outcomes of each patient (between March 15 and 23, 2020).

### Data Sources

In this study, we used patient reports from Weibo to conduct the analysis. Weibo is a Chinese microblogging website that resembles a hybrid of Twitter and Facebook; it uses a format similar to that of Twitter. This microblog provides a platform for patients infected with COVID-19 in Wuhan to seek help on the internet. Many patients reported their onset of symptoms, listed their symptoms, and uploaded their medical records and computed tomography (CT) images to seek medical care on the internet. We carefully monitored reports of patients infected with COVID-19 on Weibo between January 20 and February 15, 2020, and downloaded the data. We extracted and coded this information and deidentified the patient information by removing their names, home addresses, and contact information. We obtained 911 original COVID-19 patient reports. We only included patients who were diagnosed by positive COVID-19 tests according to the guidance provided by the Chinese National Health Commission. The positive tests consisted of either CT or real-time reverse transcriptase–polymerase chain reaction (RT-PCR) reports on the internet and were further confirmed by the follow-up telephone call.

The Weibo messages were posted from February 3 to 15, 2020. The median time from onset of symptoms to the posting of messages was 7 days (IQR 3-11). We conducted telephone follow-up calls 1 month later (between March 15 and 23) to check the clinical outcomes of the patients and acquire information about their smoking behavior, admission time, time to discharge or death, duration of time in the hospital, name of the hospital, and medication used for hypertension. Only patients who had a definite outcome (died or recovered) were included in our study.

### Data Coding

All Weibo messages were collected on the internet from February 3 to February 15, 2020. The research team extracted vital information from individual patients’ reports on the internet. A series of individual-level patient data, including demographic information, underlying comorbidities, symptoms, and signs, were coded. We double-checked and reviewed the data. The data were entered into a computerized database and cross-checked.

The symptoms coded included hypoxemia, inability to eat, cough, acute respiratory distress (ARD), dizziness, headache, confusion, unconsciousness, hemoptysis, chest pain, muscle pain, fatigue, vomiting, chest distress, loss of appetite, diarrhea, shortness of breath, and fever. The underlying comorbidities coded included chronic respiratory disease, chronic liver disease, hypertension, diabetes, chronic vascular disease, chronic lung disease, chronic heart disease, cancer, and kidney disease. Other information coded from Weibo messages included symptom onset date, sex, and age. In the telephone survey, we specifically asked which hypertension medications the patients were taking. We coded the type of medication as angiotensin-converting enzyme inhibitors (ACEIs), angiotensin II receptor blockers (ARBs), and others.

All CT images in the original posts were extracted and recorded. The CT images and CT reports were evaluated by an experienced radiologist (WJ) who was blinded to the patient survival results when she interpreted the images. The radiologist examined and coded the following features: lesion distribution, lesion characteristics, and pleural effusion.

Our data have been made public so that readers can replicate our analysis. The data can be found in the supplemental materials of this paper (Multimedia Appendix 1).

### Statistical Analysis

Descriptive statistics were obtained for all study variables. Categorical variables were described as frequency rates and percentages and were compared for the outcomes of the study using the Fisher exact test. The continuous variables were described using the median, range, and SD values and were compared using *t* tests.

Cumulative rates of death were determined using the Kaplan-Meier method. The associations between age groups, hospital admission status, lesion distribution, and pleural effusion and death outcomes were examined using the Cox proportional hazard regression model. We used the Kaplan-Meier method to plot the survival curves and used multivariate Cox regression to determine the independent risk factors for mortality.

All statistical tests were 2-tailed. *P* values <.05 were considered significant. All analyses were performed in R (R Foundation).

## Results

### Key Time-Course Distribution

We enrolled 599 patients in our study; of these patients, 516 (86.1%) recovered, and 83 (14.9%) died.

The median time from symptom onset to discharge was much longer than the time to death, namely 36 days (IQR 29.0–44.0) and 14 days (IQR 9.0-20.0, *P*<.001), respectively. The median time from the onset of symptoms to hospital admission was 11 days (IQR 7.0-15.0) for the discharged patients, compared with 10 days (IQR 6.0-13.0) for the patients who died (*P*=.70). The median time of hospital stay was 25 days (IQR 18.0-32.0) for the discharged patients and was significantly shorter for the patients who died (6 days, IQR 3.0-12.0; *P*<.001). The median time from onset of symptoms to death for patients without hospital admission was 13 days (IQR 7.0-16.75), which was not significantly different from that of patients who died in hospital (*P*=.34).

### Age and Gender

**Figure 1:**
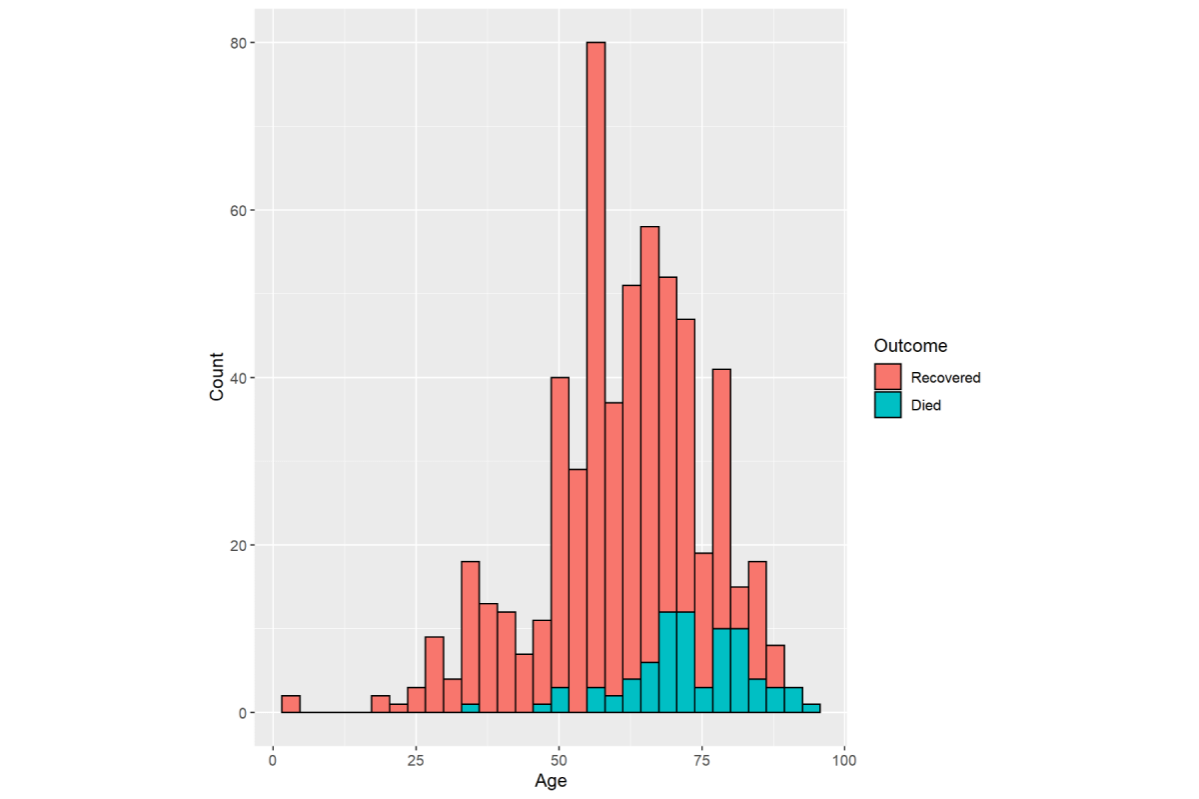
Age distributions of patients with coronavirus disease in our study who recovered and who died.

The median age of the deceased patients (72 years, IQR 66.5-82.0) was significantly higher than that of the recovered patients (61 years, IQR 53-69, *P*<.001); see Table 1 and Figure 1. Female sex was more prevalent among patients who recovered (243/516, 47.1%) than among those who died (31/83, 37.3%, *P*=.05); see Table 1.

**Table 1:** Baseline characteristics of patients with coronavirus disease (N=599).

Characteristic		Patient outcomes						*P* value	
			All (N=599)		Recovered (n=511)		Died (n=83)		
Age (years), median (range)		63 (2-93)		61 (2-89)		72 (33-93)		<.001	
Female sex, n (%)		274 (46.1)		243 (47.6)		31 (37.3)		.10	
Smoker, n (%)		123 (22.0)		106 (21.6)		17 (24.6)		.54	
**Signs and symptoms before admission, n (%)**									
	Fever		471 (79.8)		408 (80.3)		63 (76.8)		.46
	Cough		241 (40.8)		216 (42.5)		25 (30.5)		.04
	Hemoptysis		11 (1.9)		10 (2.0)		1 (1.2)		>.99
	Dyspnea		291 (49.3)		244 (48.0)		47 (57.3)		.12
	Shortness of breath		74 (12.5)		68 (13.4)		6 (7.3)		.15
	Fatigue		199 (33.7)		175 (34.4)		24 (29.3)		.38
	Muscle ache		19 (3.2)		19 (3.7)		0 (0)		.09
	Diarrhea		80 (13.6)		73 (14.4)		7 (8.5)		.17
	Chest pain		13 (2.2)		12 (2.4)		1 (1.2)		>.99
	Vomiting		72 (12.2)		67 (13.2)		5 (6.1)		.07
	Chest distress		64 (10.8)		61 (12.0)		3 (3.7)		.02
	Loss of appetite		156 (26.4)		130 (25.6)		26 (31.7)		.28
	Inability to eat		24 (4.0)		18 (3.5)		6 (7.2)		.13
	Hypoxemia		33 (5.6)		23 (4.5)		10 (12.2)		.02
	Confusion		17 (2.9)		12 (2.4)		5 (6.1)		.07
	Unconsciousness		15 (2.5)		12 (2.4)		3 (3.7)		.45
	Dizziness		18 (3.1)		16 (2.4)		2 (3.1)		>.99
	Headache		22 (3.7)		20 (3.9)		2 (2.4)		.76
**Underlying illness, n (%)**									
	Hypertension		87 (14.7)		79 (15.5)		8 (9.8)		.24
	Diabetes		57 (9.6)		49 (9.6)		8 (9.8)		>.99
	Chronic heart disease		70 (11.8)		61 (12.0)		9 (11.0)		.49
	Chronic lung disease		25 (4.1)		22 (4.3)		3 (3.7)		>.99
	Cerebrovascular disease		15 (2.5)		13 (2.5)		2 (2.4)		.95
	Chronic kidney disease		28 (4.7)		26 (5.1)		2 (2.4)		.41
	Chronic liver disease		14 (2.7)		0 (0)		14 (2.4)		.13
	Chronic respiratory disease		12 (2.0)		11 (2.2)		1 (1.2)		>.99
	Cancer		17 (2.9)		16 (3.1)		1 (1.2)		.49

### Symptoms and Clinical Characteristics

At baseline, the most common symptoms were fever (471/599, 79.8%) and cough (241/599, 40.8%); see Table 1. According to the Fisher exact test, cough and chest distress were less frequent among patients who died (25/83, 30.5%, and 3/83, 3.7%, respectively) than among recovered patients (216/511, 42.5%, and 61/511, 12.0%, respectively). The incidence rates of muscle ache and vomiting were similar between patients who died and who recovered; these differences were only marginally significant (*P*=.09 and *P*=.07, respectively). On the other hand, hypoxemia and confusion were more frequent among patients who died (10/83, 12.2%, and 5/83, 6.1%) than among patients who recovered (23/511, 4.5%, and 12/511, 2.4%; *P*=.02 and .07, respectively).

**Figure 2:**
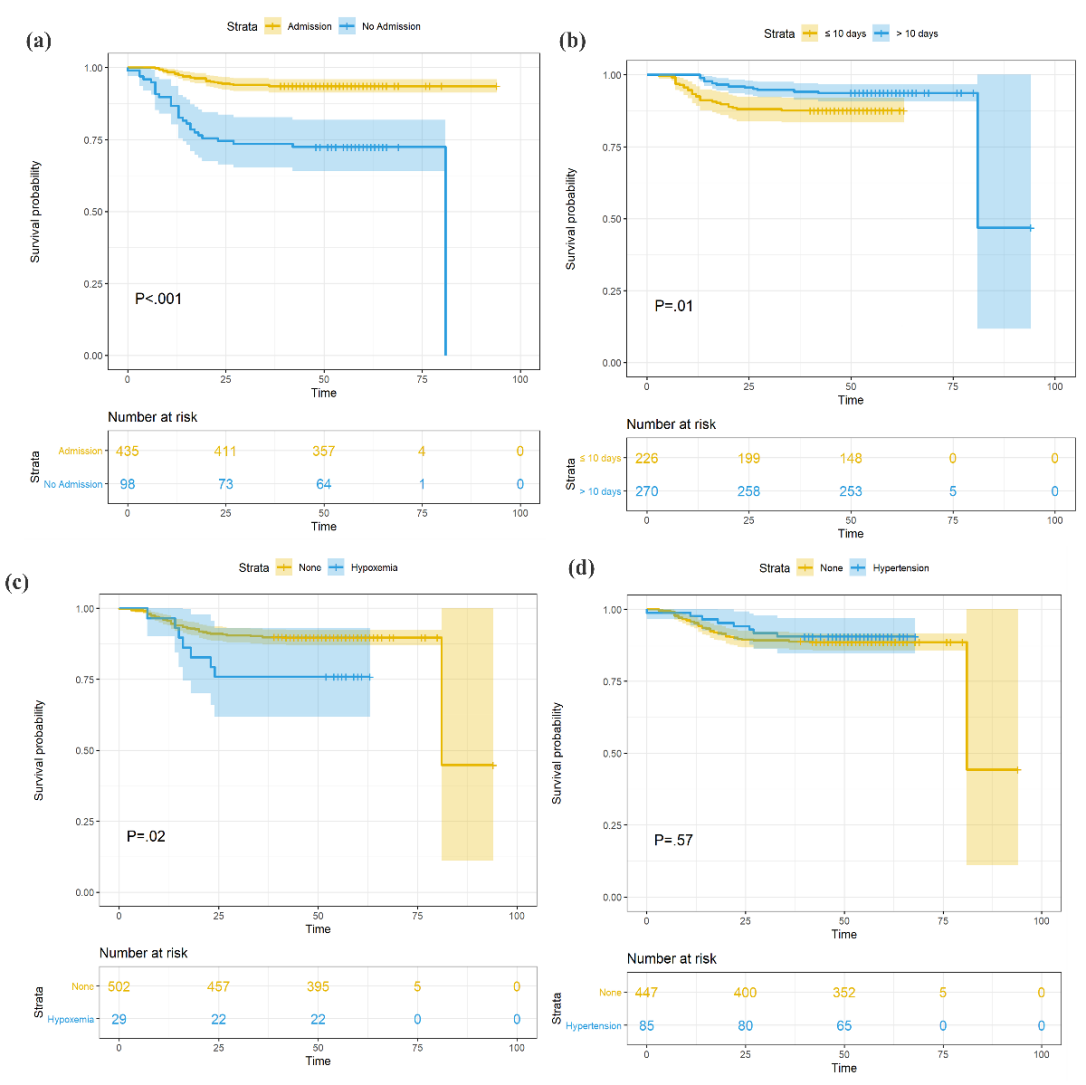
Cumulative incidence of death of patients with coronavirus disease grouped by (a) hospital admission, (b) time length between symptom onset and hospital admission, (c) hypoxemia, and (d) hypertension.

**Figure 3:**
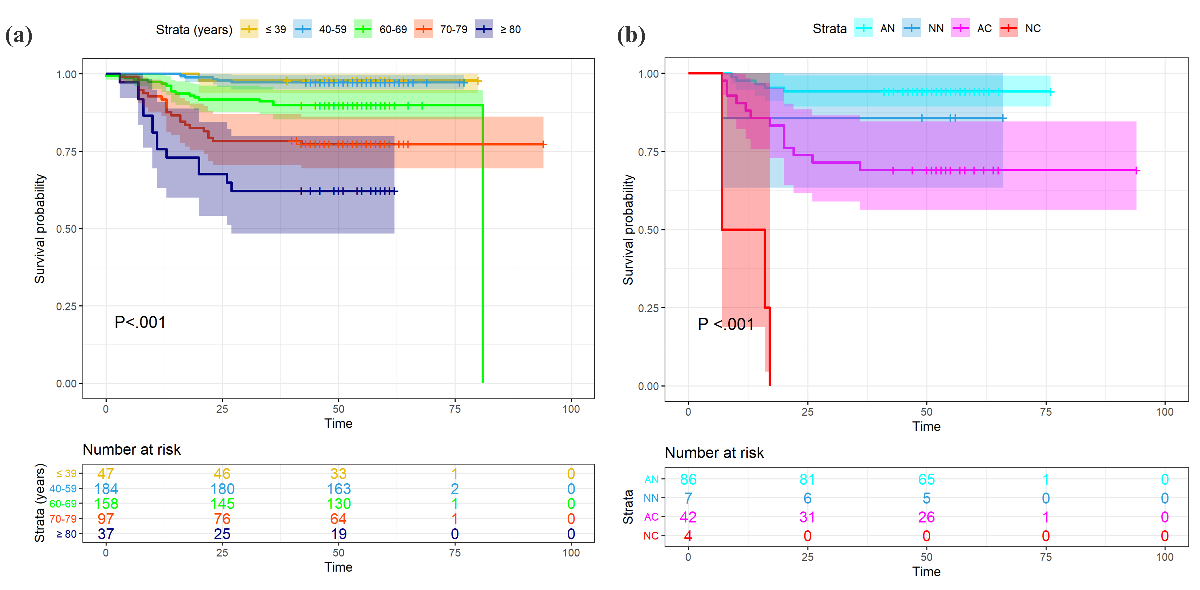
Cumulative incidence of death of patients with coronavirus disease grouped by (a) age group and (b) hospital admission and severity of illness. AC: admission, critically ill; AN: admission, not critically ill; NC: no admission, critically ill; NN: no admission, not critically ill.

Univariate Cox regression analysis showed that age older than 70 years (*P*=.02 for patients aged 70 to 79 years; *P*=.002 for patients aged ≥80 years) and hypoxemia (*P*=.03) were positively associated with death. Kaplan-Meier analysis in different groups using a log-rank test also revealed a significantly higher mortality rate for patients aged 70 to 79 years and ≥80 years and patients with hypoxemia (*P*<.001 and *P*=.02, respectively). Additionally, both univariate Cox regression and Kaplan-Meier analysis showed a lower risk of death in patients admitted to hospital (*P*<.001 for both analyses); see Table 2, Figure 2, and Figure 3.

**Figure 4:**
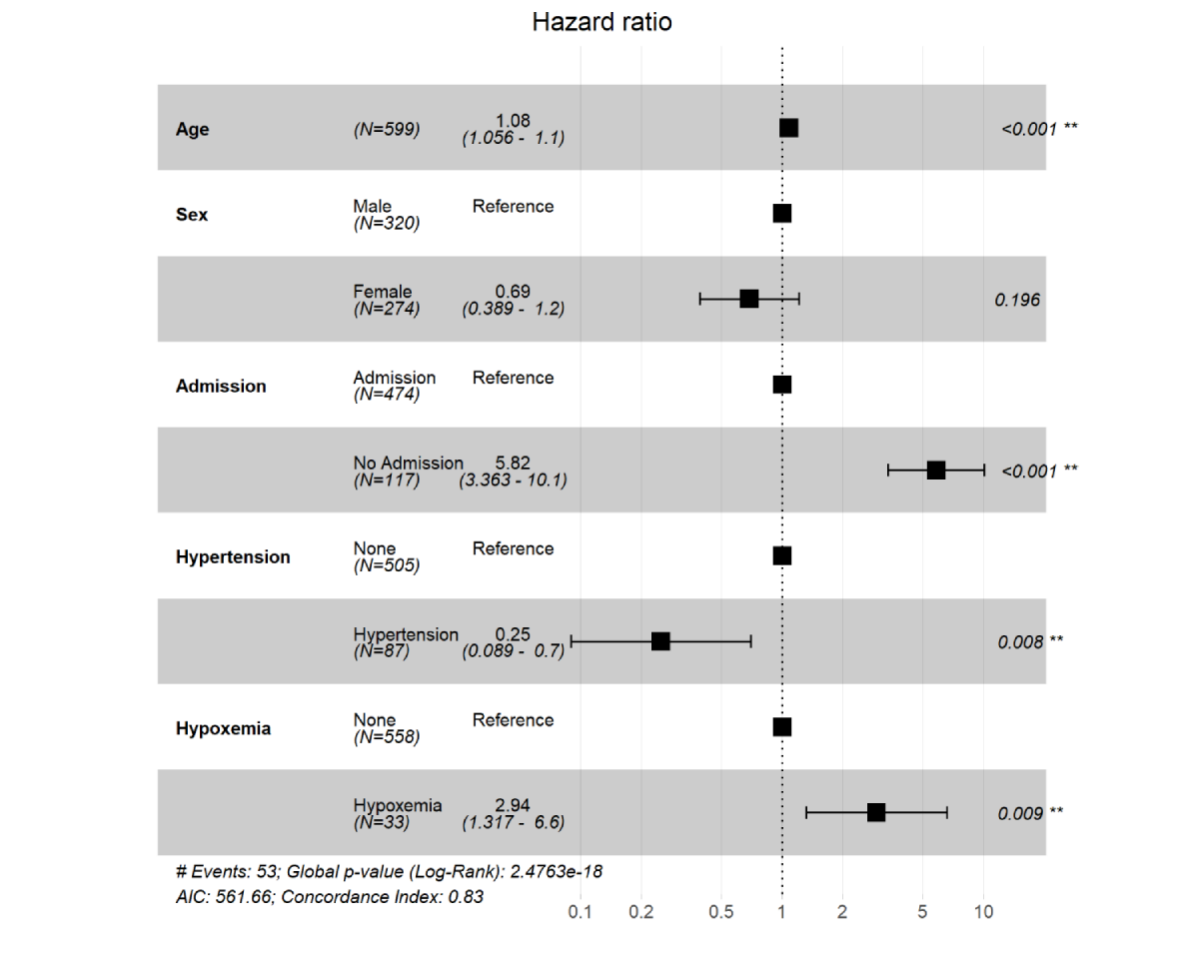
R software output showing the association of computed tomography characteristics with outcomes of patients with coronavirus disease. The hazard ratios of each variable were obtained using proportional hazard Cox models after adjustment for age and sex. **P<.01.

**Figure 5:**
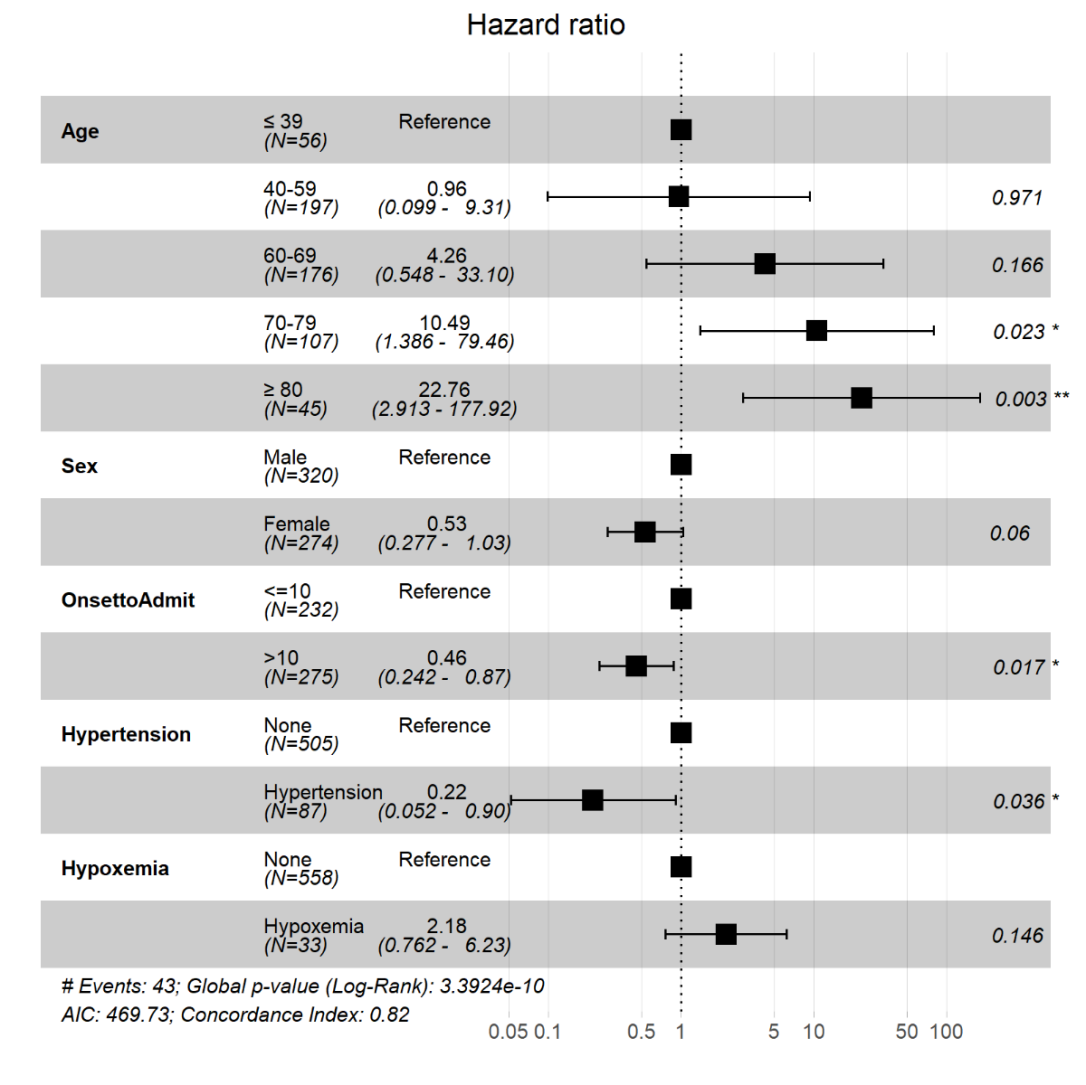
R software output showing the association of computed tomography characteristics with outcomes of patients with coronavirus disease adjusted for time between onset of symptoms and hospital admission. The hazard ratios of each variable were obtained using proportional hazard Cox models after adjustment for age and sex. Onset to admission refers to the number of days between symptom onset and hospital admission. OnsetToAdmit: onset to admission. *P<.05, **P<.01.

The multivariable-adjusted Cox proportional hazard regression model showed a significantly lower risk of death in patients with hypertension when controlling for age and sex (*P*=.008); see Figure 4. The number of days between symptom onset and hospital admission was significantly shorter in the deceased patients (*P*=.02); see Table 1 and Figure 5. Here, we adjusted the model using only age and sex because all other covariates, including symptoms and underlying diseases, were not significant and were excluded from the final model.

**Table 2:** Multivariate Cox regression analysis of the risk factors associated with mortality in patients with coronavirus disease (n=83).

Factor		Death (%)	Univariate model		Multivariate model	
			Crude hazard ratio (95% CI)	*P* value	Adjusted hazard ratio (95% CI)	*P* value
**Age (years)**						
	40-59	9 (4.6)	1.28 (0.15-10.93)	.82	1.82 (0.21-15.78)	.59
	60-69	22 (12.5)	5.19 (0.69-39.04)	.11	8.27 (1.07-63.63)	.04
	70-79	25 (23.4)	11.7 (1.58-86.87)	.02	14.01 (1.85-105.83)	.01
	≥80	21 (46.7)	22.92 (3.01-174.35)	.002	36.14 (4.68-279.27)	.001
Female sex		31 (11.3)	0.74 (0.44-1.25)	.26	0.71 (0.40-1.26)	.24
Hospital admission		33 (7.0)	0.20 (0.12-0.34)	<.001	0.16 (0.093-0.28)	<.001
Hypertension		8 (9.2)	0.80 (0.38-1.69)	.57	0.24 (0.08-0.67)	.006
Hypoxemia		10 (30.3)	2.45 (1.11-5.38)	.03	3.39 (1.51-7.62)	.003

**Radiographic Findings**

The proportions of the 227 patients with unilateral, bilateral, or diffuse pneumonia were 25 (11%), 198 (87.2%), and 4 (1.8%), respectively. Of the 224 patients with positive or negative pleural effusion, 5 (2.2%) had positive effusion; see Table 3.

**Figure 6:**
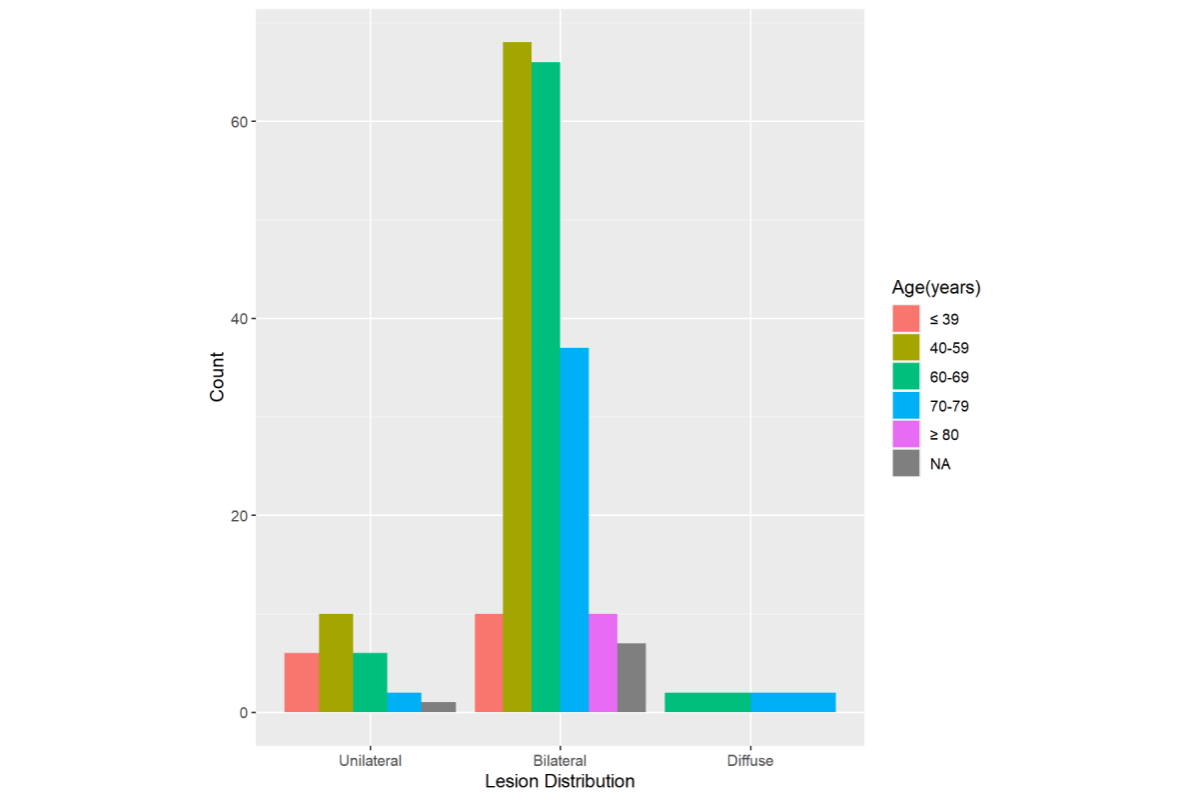
Histogram of lesion distribution among patients with coronavirus disease. NA: not applicable.

The Fisher exact tests suggested that lesion distribution differences were significant between patients who died and recovered (*P*=.045). Of the 25 patients with unilateral lesion distribution, 1 (4.0%) died. The proportions of patients in the bilateral and diffuse groups who died increased to 34/198 (17.2%) and 2/4 (50.0%); see Figure 5. In the pleural effusion positive group, 2/5 patients (40%) died. The proportion of patients in the negative group who died was 33/219 (15.1%, *P*=.18); see Table 2 and Figure 6.

**Figure 7:**
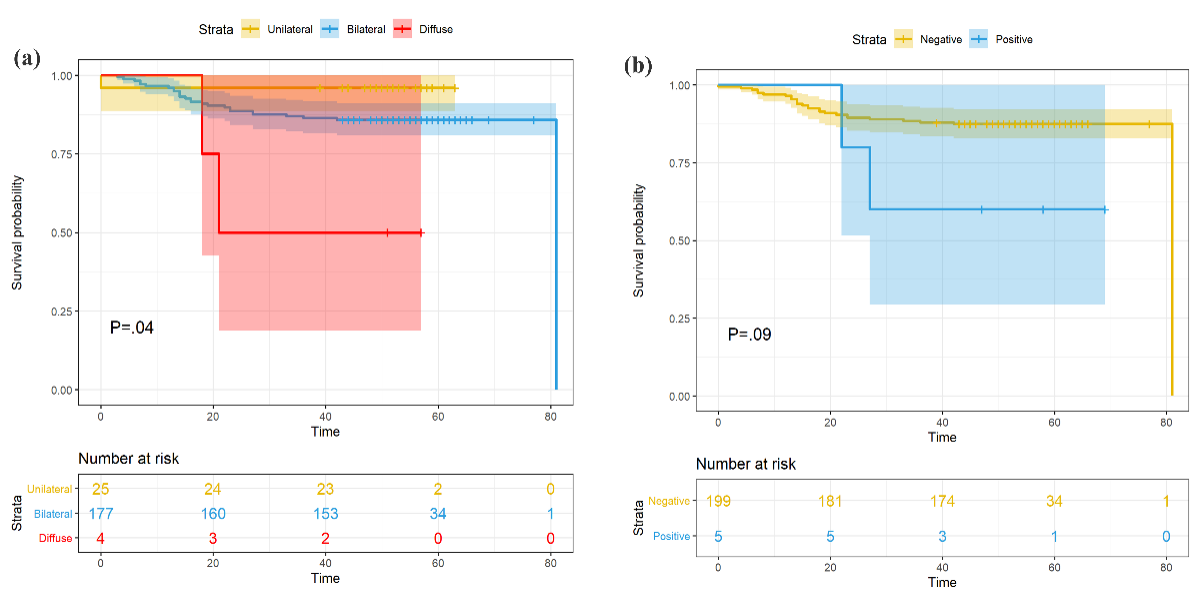
Cumulative incidence of death of patients with grouped by (a) lesion distribution and (b) pleural effusion.

**Figure 8:**
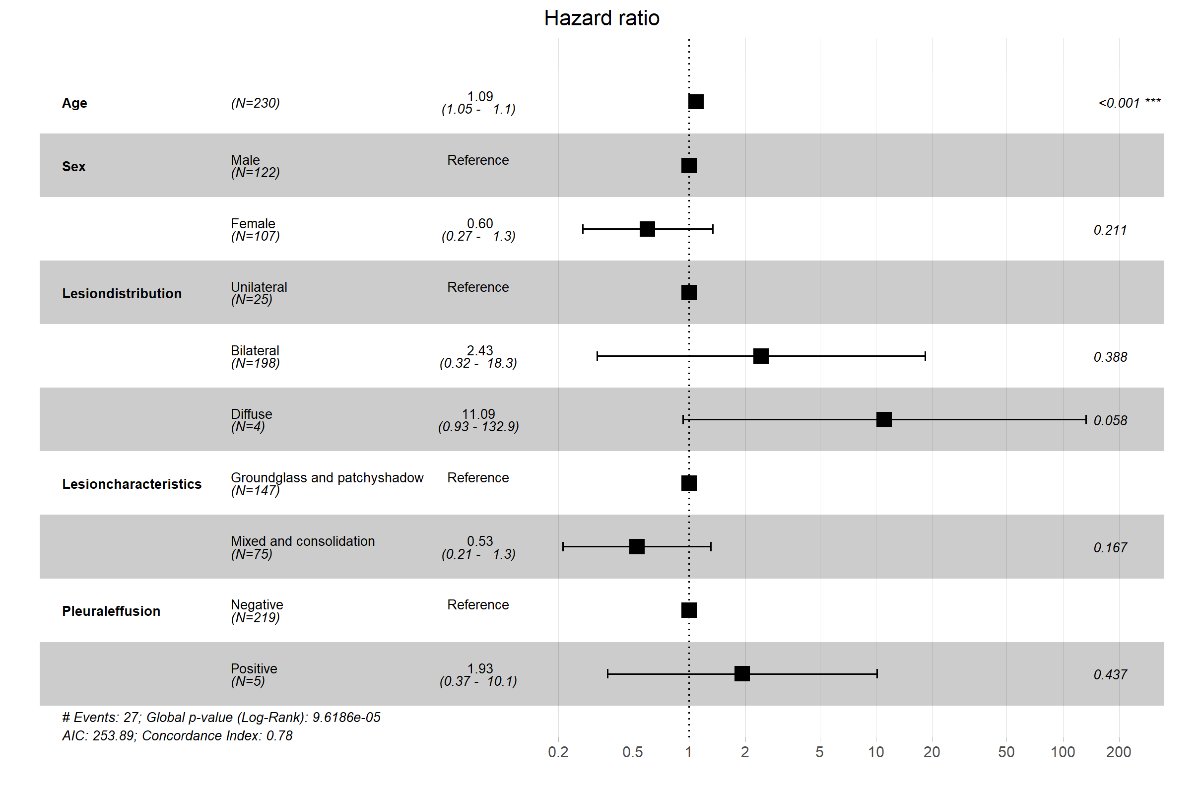
R software output showing the association of computed tomography characteristics with outcomes of patients with coronavirus disease. The hazard ratios of each variable were obtained using proportional hazard Cox models after adjustment for age and sex. ***P<.001.

**Figure 9:**
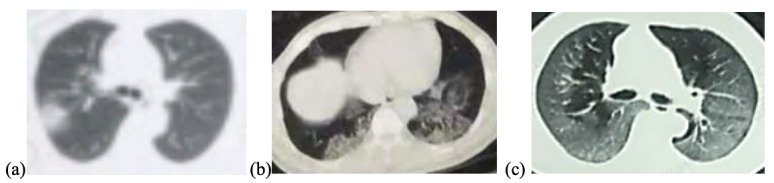
Lesion distribution on computed tomography (CT) images in patients with coronavirus disease pneumonia. (a) 60-year-old woman, unilateral lesion distribution; the axial CT image shows patchy shadowing in the right upper lobe. This patient recovered after 20 days of treatment in hospital. (b) 53-year-old man, bilateral lesion distribution; the axial CT image shows mixed lesions with ground-glass opacity and patchy shadowing in the bilateral lower lobes. This patient recovered after 30 days of treatment in hospital. (c) 68-year-old man, diffuse lesion distribution; the axial CT image shows ground-glass opacity in bilateral lungs. This patient died without treatment in hospital.

Univariate Cox regression and Kaplan-Meier analysis also showed that patients with diffuse pneumonia had a significantly higher risk of death (*P*=.02 and *P*=.04, respectively); see Figure 7. Multivariate Cox regression adjusted for age and sex showed that only diffuse pneumonia was marginally significant (*P*=.05); see Figure 8. Kaplan-Meier analysis indicated that pleural effusion was marginally significant, and it was no longer significant when multivariate Cox regression was applied. Figure 9 shows examples of lesion distributions in CT images of patients with COVID-19 pneumonia.

**Table 3:** Radiographic characteristics of patients with coronavirus disease (n=227), n (%).

Computed tomography characteristic		Patient outcomes			*P* value
		All	Recovered	Died	
**Lesion distribution**					.045
	Total	227 (100.0)	190 (83.7)	37 (16.3)	
	Unilateral	25 (11.0)	24 (12.6)	1 (2.7)	
	Bilateral	198 (87.2)	164 (86.3)	34 (91.9)	
	Diffuse	4 (1.8)	2 (1.1)	2 (5.4)	
**Lesion characteristics**					.78
	Total	222 (97.8)	186 (83.8)	36 (15.9)	
	Ground-glass opacity	120 (54.1)	100 (53.8)	20 (55.6)	
	Patchy shadowing	27 (12.2)	22 (11.8)	5 (13.9)	
	Mixed	70 (31.5)	59 (31.7)	11 (30.6)	
	Predominant consolidation	5 (2.3)	5 (2.7)	0 (0)	
**Pleural effusion**					.18
	Total	224 (98.7)	189 (83.3)	35 (15.4)	
	Negative	219 (97.8)	186 (98.4)	33 (94.3)	
	Positive	5 (2.2)	3 (1.6)	2 (5.7)	

## Discussion

### Principal Findings

In this case series of COVID-19 patients reported on the internet in Wuhan, China, one-third were older people; people aged ≥70 years represent 174/581 (29.9%) of cases. The overall mortality rate was 83/599 (13.9%). Age is one of the most frequently reported prognostic factors in COVID-19; this has been reported consistently in many recent studies worldwide [7-9]. When we stratified the data by age group, the case-fatality rate increased with age; see Figure 3. Mortality was 0/19 (0%) for patients younger than 30 years and 21/45 (47%) for patients older than 80 years. This result was similar to that in previous reports from Italy, China, and the United States [7-9]. The fatality rate was slightly higher in male patients than in female patients in this sample but was only marginally significant. Our results also showed that smoking was not associated with higher mortality risk (*P*=.54). We also added smoking to the multivariate Cox regression analysis; however, this result was also not significant.

The median time from symptom onset to discharge and the length of hospital stay was much longer than those in the deceased patients, which may be due to the discharge standard of COVID-19 patients in China (afebrile for >3 days; improved respiratory symptoms; pulmonary imaging shows apparent absorption of inflammation; two consecutive negative nucleic acid tests for respiratory tract pathogen with a sampling interval ≥24 hours) [10]. The median time length of the patients’ hospital stays was 13 days. Even when intensive and supportive care is provided, rapid progression can result in high mortality shortly after admission [11].

The typical clinical characteristics presented here were consistent with recent studies, except that the incidence of cough was lower (40.8% vs 72.2%) [12]. Because our data were acquired from the internet and were mostly reported by family members rather than the patients, some symptoms may not have been adequately presented. The incidence rate of dyspnea was found to be significantly higher in patients who died of COVID-19 in two previous reports [13,14]; this rate was also higher among patients who died in this study, but the difference was not significant. Consistent with earlier reports, hypoxemia and confusion were more frequent among patients who died [13,14]; these symptoms can be used as indicators of poor prognosis in patients with COVID-19 at baseline. It should be noted that hypoxemia was still significantly associated with death when age and sex were controlled in the multivariable-adjusted Cox regression model. On the other hand, we found that cough, vomiting, and chest distress were more frequent among recovered patients. However, these symptoms should be further confirmed before being used as favorable prognostic factors in patients with COVID-19 because of the nature of these data.

As in other reports [7,9], hypertension was the most common comorbidity in the sample. Although neither Kaplan-Meier analysis nor univariate Cox regression yielded significant results, patients with hypertension had a lower mortality risk when covariates were controlled. Several studies have shown that hypertension is related to higher mortality risk [3,15]; however, neither of these studies controlled confounding variables. Zhang et al [16] found that taking ACEIs or ARBs was associated with lower mortality risk after adjusting for covariates among patients with COVID-19 who had hypertension. The effects of ACEIs and ARBs may be positive [16,17]. Unfortunately, the data of ACEI and ARB use were not collected at first. Only 47/86 (54.7%) of the patients with hypertension answered our medication question. Of these patients, 16/47 (34%) took ACEIs or ARBs; see Multimedia Appendix 1. Although our findings suggest that hypertension and associated medication play roles in good outcomes of patients with COVID-19, their prognostic value cannot be fully verified by our study.

The predominant patterns of abnormality observed were bilateral opacity (198/227, 87.2%) and ground-glass opacity (120/222, 54.1%), which is consistent with another report [18]. We identified diffuse pneumonia as a risk factor for mortality and pleural effusion [19]. Because the CT images in our study were acquired from pictures on the internet and some were not of high quality, these findings should be further confirmed.

Hospital admission and the time from illness onset to hospitalization were significant prognostic factors. Both univariate Cox regression and Kaplan-Meier analysis indicated that hospital admission and disease severity (critically ill or not) were associated with death risk (*P*<.001). This suggests that in-hospital care can not only help patients control symptoms but can also prevent aggravation of the disease for some patients; see Figure 1 (a) and Figure 4. Although the time from illness onset to hospitalization was not significantly longer in the death group, the Kaplan-Meier analysis of the time from illness onset to admission (≤10 days or >10 days) implied that patients with delayed medical care had a higher mortality risk; see Figure 1 (b). Together, these findings suggest that timely hospital care contributes to alleviating the severity and improving the prognosis of patients with COVID-19; see Figure 1 (e).

**Figure 10:**
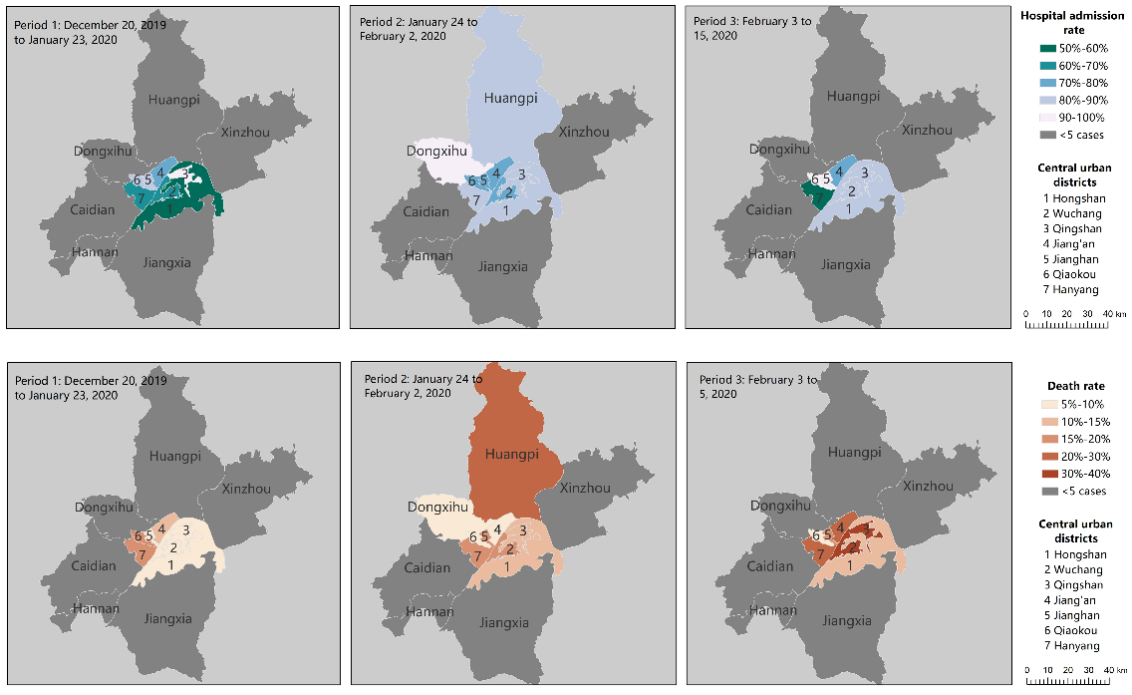
Geographic distributions of mortality and hospital admission rates of coronavirus cases across three time periods in Wuhan, China. The mortality and admission rate of the cases were calculated using the number of deaths or admissions divided by the total number of cases reported in the area and time period. The data include all 13 districts of the city of Wuhan; regions with fewer than 5 cases were considered to be nonrepresentative and are plotted in grey.

We also plotted the mortality and admission rates of COVID-19 on the map across three periods. According to Pan et al [20], after outbound transportation from Wuhan was blocked and public and vehicular transit were suspended on January 23, 2020, the effective reproduction number (R_t_) of COVID-19 was reduced and its spread to other cities was delayed [21]. However, previous reports did not evaluate the effects of city shutdown on hospital admission and death rates. Figure 10 shows the geographic distributions of the mortality and hospital admission rates of COVID-19 cases across three time periods in Wuhan. The three time periods depicted are in reference to Pan et al [20]. The time from December 20, 2019, to January 23, 2020, was considered as the first period, when no COVID-19–specific interventions were imposed. After January 23, the government blocked all outbound transportation from Wuhan and suspended public and vehicular transportation in the city. On February 2, centralized quarantine and a “treatment of all cases” policy were implemented, and the number of hospital beds and medical supplies increased. We found that the mortality rate rose in most districts of Wuhan following the closure of the city. On the other hand, the admission rates fluctuated in different regions in periods 2 and 3. There were noticeable geographic differences in mortality rates, with the highest rates occurring in the suburban districts (see Multimedia Appendix 1). The shutdown of the city may have helped control the disease; however, it may have led to a rise in the mortality rate in a certain period. It should be noted that this result should be explained with caution, as our sample was not random and may not be representative.

### Policy Implications

Our study suggests that use of social media data can be effective to identify patients at high risk for COVID-19, help coordinate appropriate treatment, and lower the mortality rate. The use of social media can also reduce cross-infection risks by reducing the number repeat visits of low-risk patients to the hospital. In the future, social media can be adopted to effectively help potentially critically ill patients seek timely medical treatment, help patients with low mortality risks to reduce unnecessary cross-infection, screen out critically ill patients in urgent need of hospitalization, and finally to facilitate disease control and hierarchical management. One limitation of this method is that it still requires particular definition and attention from the aspects of law, policy, and ethics; another limitation is that it requires active management and supervision procedures with participation of medical professionals to ensure its accuracy, effectiveness, and reasonableness.

### Limitations

This study has several limitations. First, the data were acquired on the internet and followed up via telephone. The nature of the data did not allow us to obtain more detailed information. Second, we did not obtain details regarding the patients’ laboratory characteristics, clinical course, or treatment. Also, some radiological files were not complete.

### Conclusions

Hospital admission at an appropriate time is vital for patients with COVID-19, especially those who are critically ill. Older age, hypoxemia, and pleural effusion were related to poor prognosis of mortality. Public health measures such as transportation blocking and city closure should be combined with other measures, such as increasing admission rates and shortening wait times for treatment.

Currently, more than 2.9 billion individuals use social media regularly. Considering the substantially high speed, reach, penetration, and transparency of social media platforms, social media can be used not only to disseminate but also to collect critical information about a sudden outbreak of disease. Individual patients’ reports of their symptoms, clinical characteristics, treatment, and clinical outcomes on social media can be aggregated into big data and analyzed in real time to provide valuable insights to accelerate research speed [22].

## Appendix

Multimedia Appendix 1 Supplemental materials.

## Abbreviations

ACEI: angiotensin-converting enzyme inhibitor
ARB: angiotensin receptor blocker
ARD: acute respiratory distress
COVID-19: coronavirus disease
CT: computed tomography
ICU: intensive care unit
R_t_: effective reproduction number
RT-PCR: reverse transcription–polymerase chain reaction
