# Supplementary material for: Characteristics and Outcomes of a Sample of Patients With COVID-19 Identified Through Social Media in Wuhan, China: Observational Study
Source: J Med Internet Res. 2020 Aug 13;22(8):e20108. doi: 10.2196/20108 (PMC7431239; doi:10.2196/20108)
Supplement: Multimedia Appendix 1 [file jmir_v22i8e20108_app1.docx]

Table 1 Hospitals and the number of patients they admitted in our sample.

| **Hospitals** | **NO of Patients** |
| --- | --- |
| Mobile Cabin Hospital | 80 |
| Tongji Medical College Huazhong University of Science &Technology | 61 |
| Wuhan Union Hospital of China | 37 |
| Fire God Mountain Hospital | 34 |
| Wuhan Jinyintan Hospital | 26 |
| The First Hospital of Wuhan | 25 |
| Renmin Hospital of Wuhan University | 20 |
| Thunder God Mountain Hospital | 17 |
| The Third Hospital of Wuhan | 16 |
| Wuhan Wuchang hospital | 15 |
| Taikang Tongji (Wuhan) hospital | 11 |
| The Sixth Hospital of Wuhan | 11 |
| The Central Hospital of Wuhan | 11 |
| The Ninth Hospital of Wuhan | 10 |
| The Fourth Hospital of Wuhan | 10 |
| Hubei Provincial Hospital of TCM | 9 |
| Wuhan Hankou hospital | 7 |
| Hubei Provincial Hospital of Integrated Chinese & Western Medicine | 5 |
| Tianyou Hospital Affiliated to Wuhan University of science and technology | 5 |
| The Fifth Hospital of Wuhan | 5 |
| Wuhan Hanyang hospital | 5 |
| Wuhan Red Cross Hospital | 5 |
| The First People's Hospital of Jiangxia | 5 |
| Wuhan Hospital of TCM | 5 |
| The Third People's Hospital of Hubei Province | 4 |
| The Seventh Hospital of Wuhan | 4 |
| Wuhan Zijing Hospital | 4 |
| The Eighth Hospital of Wuhan | 3 |
| Wuhan second Chinese & Western Medicine Hospital | 3 |
| The People's Hospital of Dongxihu | 3 |
| Wuhan Pulmonary Hospital | 3 |
| Huangpi District Hospital of TCM | 3 |
| Wuhan Asia General Hospital | 3 |
| Wuhan Asia Heart Hospital | 3 |
| Union Jiangbei Hospital | 2 |
| The Second Hospital of Wisco | 2 |
| Wuhan Puren Hospital | 2 |
| Wuhan Business Professionals Hospital | 2 |
| Central Hospital of Central theater Command | 2 |
| Ezhou Third Hospital | 1 |
| Ezhou Hospital of Traditional Chinese Medicine | 1 |
| Maternal and Child Hospital of Hubei Province | 1 |
| CR&WISCO General Hospital | 1 |
| Liyuan Hospital of Tongji Medical College of Huazhong University of Science &Technology | 1 |
| Nanzhang County People's Hospital | 1 |
| The First People's Hospital of Tianmen in Hubei Province | 1 |
| Tongpu hospital | 1 |
| Wuhan Women and Children Medical Care Center | 1 |
| Hubei General Hospital Medical Conjoined Wuhan Jihe Hospital | 1 |
| Wuhan Ruihua Hospital | 1 |
| Dongxihu District Jinghe Health Center of Wuhan | 1 |
| The People's Hospital of Huangpi | 1 |
| Wuhan Jiangjun Road Community Hospital | 1 |
| The People's Hospital of Xinzhou | 1 |
| General Hospital of the Yangtze River Shipping / Wuhan Brain Hospital | 1 |

Table 2 Hypertension Medicine Lists

| **Hypertension Medicine** | **Count** | **ACEI/ARB（1=Yes; 0=No）** |
| --- | --- | --- |
| Amlodipine Besylate and Benazepril Hydrochloride Tablets(I) | 1 | 1 |
| Amlodipine Besylate Tablets | 10 | 0 |
| Amlodipine Maleate Dispersible Tablets | 1 | 0 |
| Amlodipine Maleate Tablets | 1 | 0 |
| Benazepril Hydrochloride Tablets | 1 | 1 |
| Captopril Tablets | 2 | 1 |
| Fosinopril Sodium Tablets | 1 | 1 |
| Indapamide Tablets | 1 | 0 |
| Irbesartan and Hydrochlorothiazide tablets | 1 | 1 |
| Levamlodiping Besylate Tablets | 5 | 0 |
| Metoprolol Succinate Sustained-release Tablets | 1 | 0 |
| Metoprolol Tartrate Tablets | 1 | 0 |
| Nifedipine Controlled-release Tablets | 3 | 0 |
| Nifedipine Sustained-release Tablets | 2 | 0 |
| Nifedipine Sustained-release Tablets(Ⅱ) | 2 | 0 |
| Nifedipine Tablets | 2 | 0 |
| Perindopril Tablets | 2 | 1 |
| Valsartan and Amlodipine Tablets(Ⅰ) | 2 | 1 |
| Valsartan Capsules | 1 | 1 |
| Levamlodiping Besylate Tablets； Valsartanand Amlodipine Tablets(Ⅰ) | 1 | 1 |
| Levamlodiping Besylate Tablets； Metoprolol Tartrate Tablets | 1 | 0 |
| Nifedipine Sustained-release Tablets； Captopril Tablets | 1 | 1 |
| Benazepril Hydrochloridec Tablets； Felodipine Sustained Release Tablets | 1 | 1 |
| Aspirin Enteric-Coated Tablets； Candesartan Cilexetil Tablets； Carvedilol Tablets； Isosorbide Mononitrate Sustained Release Tablets | 1 | 1 |
| Amlodipine besylate tablets；Spironolactone Tablets； Valsartan and Amlodipine Tablets; Terazosin Hydrochloride Tablets；Atorvastatin Calcium Tablets；Metoprolol Succinate Sustained-release Tablets | 1 | 1 |
| Levamlodiping Besylate Tablets； Metoprolol Tartrate Injection； Levamlodipine Besylate Tablets | 1 | 0 |


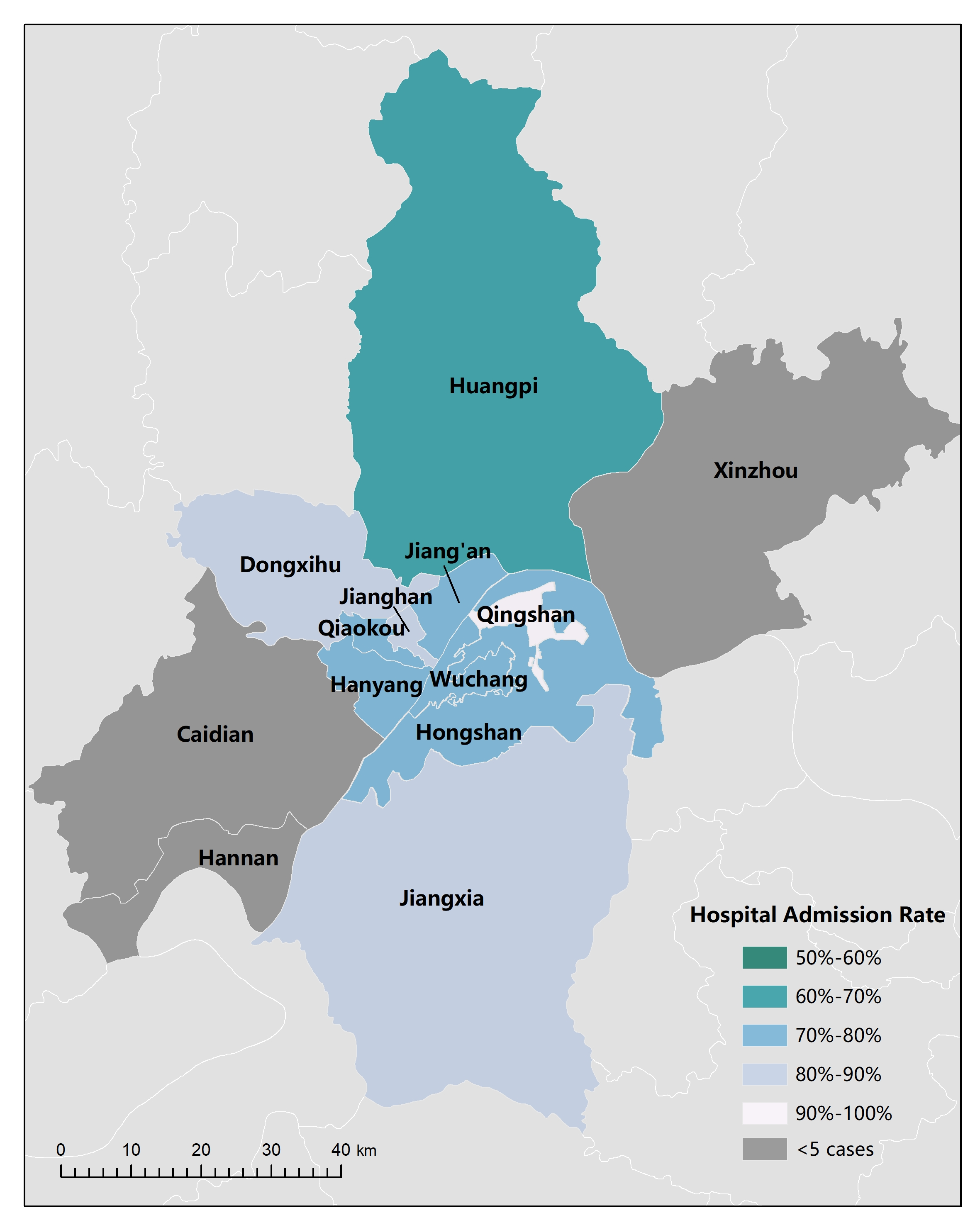


Figure 1 The Geographic Distribution of Admission Rates of COVID-19 Cases in Wuhan, China.


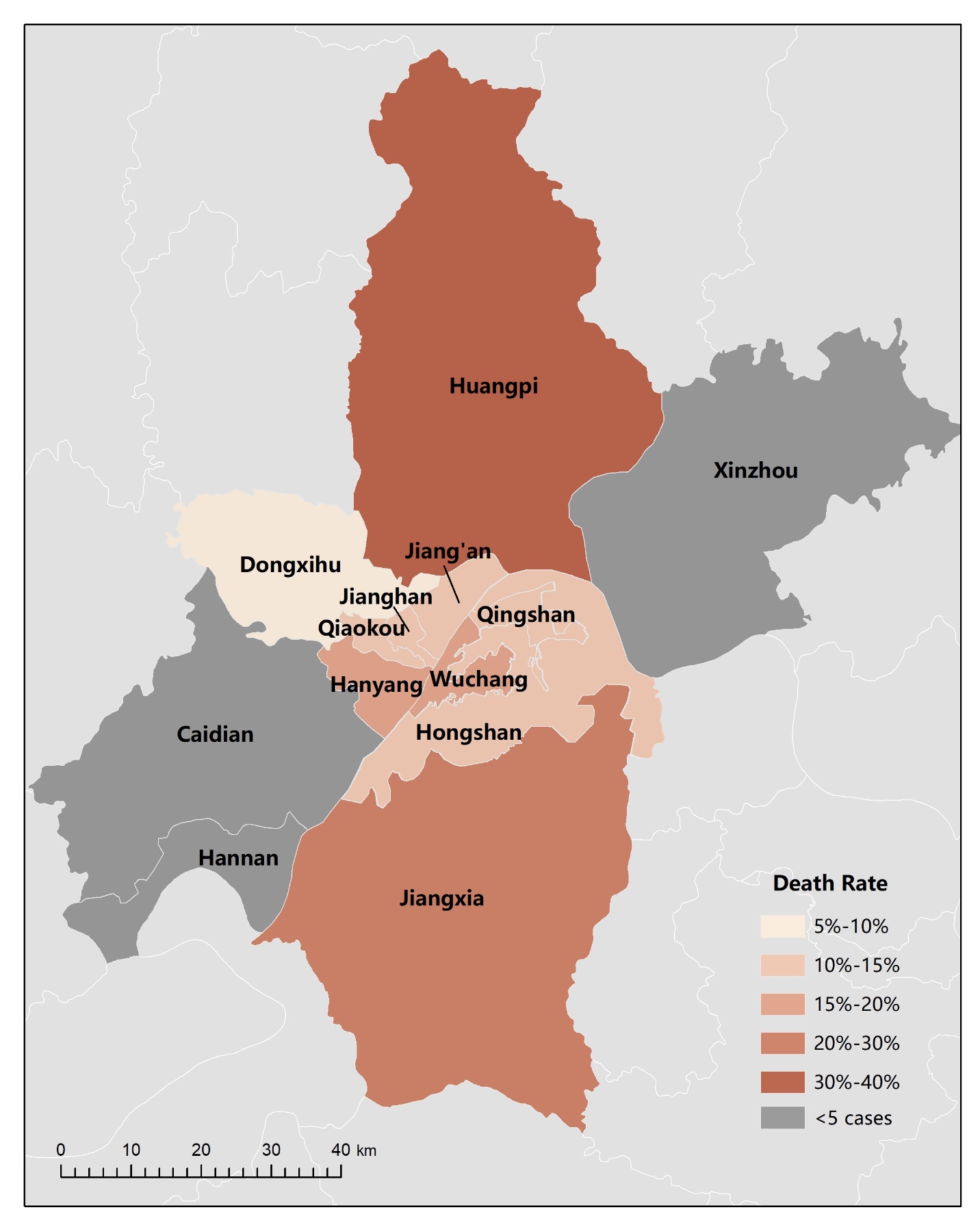


Figure 2 The Geographic Distribution of Moratlity Rates of COVID-19 Cases in Wuhan, China.
